# Effects of climate and fire on short-term vegetation recovery in the boreal larch forests of Northeastern China

**DOI:** 10.1038/srep37572

**Published:** 2016-11-18

**Authors:** Zhihua Liu

**Affiliations:** 1 Key Laboratory of Forest Ecology and Management, Institute of Applied Ecology, Chinese Academy of Sciences, Shenyang, 110164, China

## Abstract

Understanding the influence of climate variability and fire characteristics in shaping postfire vegetation recovery will help to predict future ecosystem trajectories in boreal forests. In this study, I asked: (1) which remotely-sensed vegetation index (VI) is a good proxy for vegetation recovery? and (2) what are the relative influences of climate and fire in controlling postfire vegetation recovery in a Siberian larch forest, a globally important but poorly understood ecosystem type? Analysis showed that the shortwave infrared (SWIR) VI is a good indicator of postfire vegetation recovery in boreal larch forests. A boosted regression tree analysis showed that postfire recovery was collectively controlled by processes that controlled seed availability, as well as by site conditions and climate variability. Fire severity and its spatial variability played a dominant role in determining vegetation recovery, indicating seed availability as the primary mechanism affecting postfire forest resilience. Environmental and immediate postfire climatic conditions appear to be less important, but interact strongly with fire severity to influence postfire recovery. If future warming and fire regimes manifest as expected in this region, seed limitation and climate-induced regeneration failure will become more prevalent and severe, which may cause forests to shift to alternative stable states.

Boreal forests comprise one third of the global forested area, and regulate the climate system via the carbon cycle and land-atmosphere energy exchange^1^,^2^,^3^. Recent unprecedented rates of climate warming and an associated increase in wildfire frequency, severity and size^4^,^5^ have the potential to push the boreal forest ecosystem across a threshold beyond which forest dieback, forest compositional change, and loss of carbon sequestration capacity may occur^6^,^7^,^8^. Accumulating evidence suggests that initial forest conditions and disturbance legacy effects can play key roles in determining postfire community composition and function in boreal forests^9^, and interactions among climate variability, historical contingency, and vegetation disturbance responses may cause threshold responses and generate unexpected ecosystem trajectories in the biome^10^. As boreal forests are increasingly affected by intensifying fire regimes and a greater degree of climatic variability, an improved understanding of the mechanisms that determine vegetation recovery following disturbance is critical for providing insight into the potential for novel ecological trajectories^11^.

Observational and experimental studies have found that fire interacts with tree species’ fire-adaptation strategies, strongly affects seed availability and site condition, and thus plays a dominant role in determining tree recruitment in the boreal forest ecosystem^12^,^13^. In fire “embracer”-dominated forests, such as those dominated by *Picea mariana*, *Pinus contorta*, and *Pinus banksiana*, fires are typically of intermediate frequency and high severity. However, forests can generally regenerate locally from seeds stored in serotinous cones^9^,^14^. As a result, site conditions (e.g., organic layer depth) play a major role in determining the postfire successional trajectory of forests due to the different regeneration abilities of deciduous hardwoods and conifers^9^,^15^. In fire “avoider”-dominated forests, where species such as *Abies sibirica*, *Abies nephrolepis*, *Picea abies* and *Picea obovata* are dominant, fire is infrequent and stand-replacing, and trees rarely survive fires. These forests rely on seed dispersal from unburned trees to regenerate. Therefore, seed limitation plays a major role in determining postfire successional trajectories via the different dispersal abilities of deciduous hardwoods and conifers^13^. In fire “resister”-dominated forests, such those where *Pinus sylvestris* and *Larix spp*. are dominant, fire is frequent and of low to medium intensity, and trees usually survive fire due to their thick bark. Although “resisters” dominate the light taiga of Siberia and cover about 20% of global boreal forests, little research has been conducted to investigate the mechanisms that determine postfire vegetation recovery.

Climate variability is another major determinant of postfire vegetation recovery^16^. Climate affects vegetation recovery directly through its influence on plant demography (i.e., colonization and establishment) and indirectly through its influence on fire regime. Plants depend on favorable climatic conditions to regenerate and survive. Plant establishment and growth are generally higher under favorable postfire climatic conditions, such as conditions with higher precipitation and moisture availability^17^,^18^. Fire regimes are largely climate-driven, and are expected to intensify with warmer and drier climates in boreal forests^1^. As a result, altered seed availability and site conditions under intensified fire regimes may slow forest recovery^19^,^20^, or change successional trajectories^9^. Furthermore, homogenization of fire severity within individual fires may alter vegetation legacies and burned patch configurations, and thus change seed dispersal patterns on the landscape^11^,^14^,^16^. Once established, the longer-term vegetation successional trajectories depend on initial vegetation density and composition^21^,^22^,^23^ and favorable climate conditions. Therefore, vegetation recovery is determined by multiple interacting factors ranging from fire characteristics, climate variability, and plant regeneration strategies, to environmental filters at variable spatial and temporal scales. Disentangling the relative influences of climate variability and fire characteristics, among other environmental filters, is challenging.

Previous studies have made substantial contributions to our understanding of the influence of site conditions and seed availability in shaping postfire successional trajectories. However, most studies are limited in space and time, and therefore have limited abilities to disentangle the effects of multiple interacting factors on vegetation recovery and generalize these at large scales. In contrast, satellite observations of postfire studies can potentially overcome these limitations through spatially and spectrally consistent monitoring of fire characteristics and fire effects on vegetation over large regions. Normalized Difference Vegetation Index (NDVI) is the most commonly vegetation index to monitor postfire vegetation recovery trajectories due to its sensitivity to vegetation chlorophyll content or “greenness”^24^,^25^. However, NDVI, like many other vegetation indices, is also limited by a number of issues, including saturation in closed canopies, and sensitivities to atmospheric aerosols and soil background^26^. Recent remote sensing analyses have shown that short-wave infrared (SWIR)-based vegetation indices are sensitive to vegetation water content and canopy structure in boreal Canadian^27^ and Siberian forests^28^. However, the potential of SWIR-based vegetation indices for measuring vegetation recovery has not yet been fully explored^29^.

The objective of this analysis is to explore the potential roles of climate variability and fire characteristics in controlling the short-term (~5 years) vegetation recovery in a “resister”-dominated Dahurian Larch (*Larix gmelini*) forest in a climate- and fire-sensitive region of Northeastern China. As fire “resisters”, Larch trees survive low to medium intensity fires, and provide abundant seed source for forest recovery. Consequently, the Larch forests are unlikely to be seed-limited except following very severe fires with few surviving seed sources^30^. The study region is in the transition zone between boreal and temperate along a temperature gradient, and between woodland and grassland zones along a moisture gradient (see *Study area*). Climate variability has an important role in determining vegetation composition and dynamics^31^. Therefore, the central hypothesis is that climate variability is the primary determinant of postfire vegetation recovery in this region. In this study, this hypothesis is tested by investigating postfire vegetation recovery with varying climatic conditions and fire characteristics across a large spatiotemporal scale using a remote sensing approach. The specific research questions are (1) to determine the best vegetation index for measuring postfire vegetation recovery, and (2) to understand the relative influences of climate and fire characteristics on postfire vegetation recovery in a Siberian Larch forest. This study will elucidate the role of changing fire regimes and climate in shaping vegetation recovery and contribute to the understanding of fire-vegetation-climate interactions in a globally important, but poorly-understood boreal forest.

## Methods

### Study area

**Figure 1:**
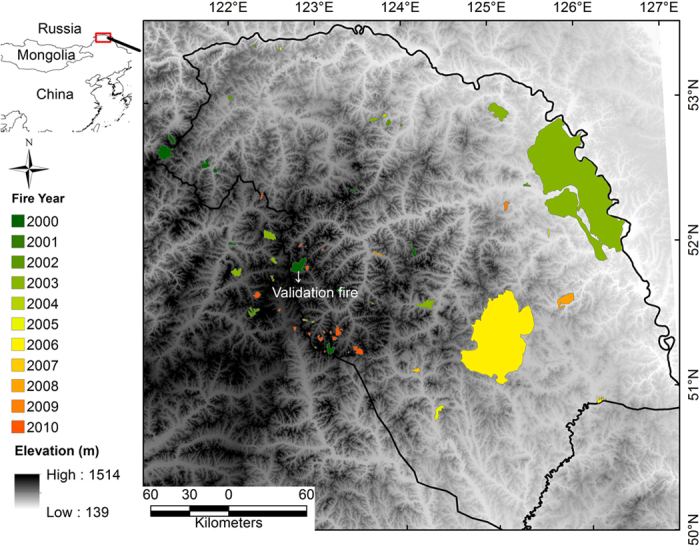
Study area with burned patches (2000–2010), and digital elevation model (DEM) map. The DEM was download from SRTM (Shuttle Radar Topography Mission) website (http://www2.jpl.nasa.gov/srtm/). Burned patches were manually digitized based on reported fire information and Landsat images (this study). The burned patch used for field measurement was also indicated. The map was produced in ArcGIS 10.3 (http://www.esri.com/).

The Great Xing’an Mountains are located in the boreal-temperate forest transition zone of Northeastern China, and encompass approximately 8.46 × 10^4^ km^2^ (Fig. 1). The area has a cold, continental climate, with average annual temperatures declining from 1 °C at the southern extreme to −6 °C at the northern extreme, and precipitation declining from 442 mm in the South to 240 mm in the North. The vegetation of this area is representative of cool temperate coniferous forests, forming the southern extension of the eastern Siberian Larch forests. The forests mainly comprise of Dahurian Larch, with a few other locally abundant species, such as Pine (*Pinus sylvestris var. mongolica*), Spruce (*Picea koraiensis*), Birch (*Betula platyphylla*), two species of Aspen (*Populus davidiana, Populus suaveolens*), Willow (*Chosenia arbutifolia*), and a shrub species, *Pinus pumila*. The historical fire regime of this eastern Siberian boreal forest included low intensity surface fires mixed with infrequent stand-replacing fires, with fire return intervals ranging from 30 to 120 years^32^. However, intensive forest management and fire suppression since the 1950s have altered fire regimes in this region, and current fires have been infrequent, but more intense, with a fire return interval of more than 500 years^33^. Understory species composition is closely related to topographic position in both mature and early post-fire successional stages^34^. Well-drained, south-facing slopes are generally warm and dry, and have mostly herbaceous species and a few shrub species, such as *Epilobium angustifolium* L., *Rubus sachalinensis* var. *sachalinensis*, *Rhododendron dauricum* L. These slopes experience fires of relatively higher frequency and lower intensity. North slopes and terraces are generally wet and cool, and dominated by two shrub species, *Ledum palustre* and *Vaccinium uliginosum* (up to 0.4 m in height), supporting relatively infrequent, high intensity fires due to more contiguous and high-loading fuels^35^. Postfire tree seedling consists mainly larch and birch, and the understory species contribute more to early vegetation recovery in higher severity burned patches^20^.

### Conceptual model and variable selection

**Figure 2:**
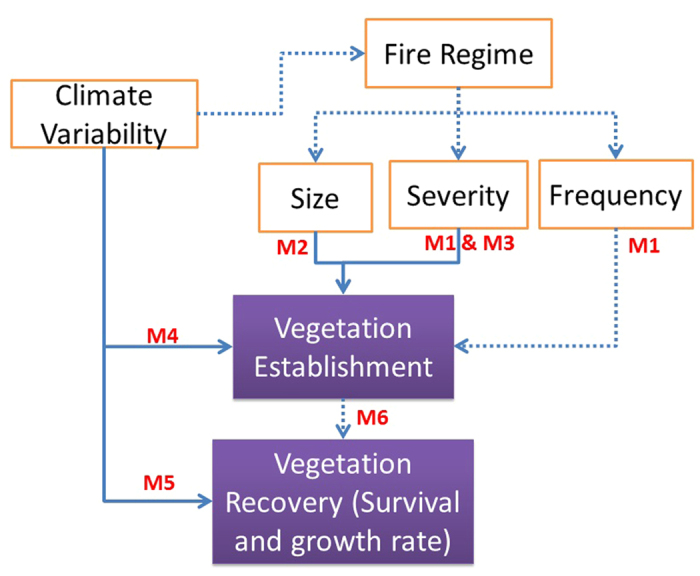
Hypothetical relationship among climate variability, fire regime, and postfire vegetation recovery in boreal forest ecosystems (see text for details). Solid lines indicated mechanisms that were tested in current analysis.

The effects of climate variability and fire characteristics on vegetation recovery in boreal forests relates to a series of mechanisms that control seed supply, seed delivery, seed regeneration, and seedling establishment and growth (Fig. 2; M1-M6)^36^. First, seed supply (M1) is related to fire frequency which alters seed production depending on the balance between fire return intervals and the time required for trees to reach maturity. Seed supply is also related to fire severity, which determines seed survival^37^. Second, seed delivery (M2) relates to the patch size of high-severity burns and to the seed dispersal abilities of different species^38^. Third, seed regeneration is determined by both seedbed environment (M3) and immediate postfire climatic conditions (M4). Fire severity modifies site conditions which may favor some tree species more than others, resulting in changes in postfire species composition. For example, increasing fire severity promotes the recruitment of deciduous tree species and decreases the relative abundance of black spruce immediately following fire in interior Alaska^9^. Postfire seedling recruitment typically occurs during a short postfire climatic window, and therefore immediate postfire climate conditions control seed regeneration and seedling survival. For example, drier years following fires have been found to suppress the seed regeneration in subalpine and montane forests in western North America^16^. Finally, vegetation recovery also depends on longer-term climate suitability (M5), and successional pathways (M6). All else being equal, favorable climatic conditions generally promote survival and growth rates. The ecosystem successional trajectory is also determined by postfire species composition and density^11^,^36^. These climate and fire mechanisms were also modulated by biophysical environment of burned patches. All these mechanisms and related variable selection was listed in Table 1.

### Dataset

#### Landsat data

**Figure 3:**
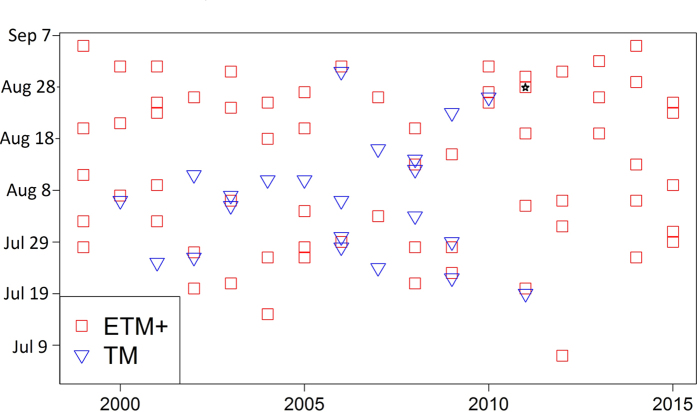
Distribution of Landsat images used to characterize the postfire vegetation recovery trajectory. The image (August 28, 2011) used for calculating vegetation indices and correlating with field data is marked with an asterisk.

Thirty-meter resolution Landsat data were used to characterize postfire vegetation recovery between 1999 and 2015. Landsat images were mostly selected from the peak growing season (July and August) and acquired by the ETM+ sensor with cloud cover <20% to minimize the potential influence of phenology, sensors, and clouds on vegetation indices^39^. If no suitable ETM+ image was found for a particular fire, ETM+ images were acquired between June 15 and 30 or July and August images were acquired from the TM sensor. Of the 94 images used in this analysis, 84 images were from the peak growing season, and 71 were from the ETM+ sensor (Fig. 3). Landsat L1T data acquired from USGS GLOVIS portal (http://glovis.usgs.gov/) has been radiometric and geometric corrected and were converted to top-of-atmosphere reflectance and then atmospherically corrected to surface reflectance using the Landsat Ecosystem Disturbance Adaptive Processing System (LEDAPS)^40^. The data gap caused by the Scan Line Corrector failure in each ETM+ images after May 31, 2003 was removed from the analysis. Clouds and cloud shadows were masked out using an object-based approach called the F-mask algorithm^41^. The potential influences of topography and sun-sensor geometry (e.g., BRDF effects) on surface reflectance were relatively minor and were not corrected^42^.

#### Fire scars

Historical burned patches between 2000 and 2010 were manually digitized based on fire ignition locations and year^5^ using delta Normalized Burn Ratio (dNBR) images. The dNBR is widely used in mapping burned patches and severity in various forest ecosystems^43^,^44^, and was proven to be applicable to our study area by comparing dNBR values with field-based burn severity data^45^. The dNBR images were calculated by subtracting NBR images one year before the fire from NBR images in the fire year, as fires usually burned prior to the peak growing season. Large positive dNBR values indicate disturbance events. The NBR is computed as:


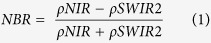


where *ρNIR* is the reflectance in the NIR wavelength, Landsat TM/ETM+ band 4 (0.76–0.90 μm) and *ρSWIR*2 is the reflectance in the SWIR wavelength, Landsat TM/ETM+ band 7 (2.08–2.35 μm).

A histogram approach and results from a previous publication^45^ were combined to determine the dNBR threshold (>0.1) to separate burned pixels from unburned pixels. A total of 84 fires, ranging from 8 to 239,500 ha with a median of 468 ha, that burned over 685,930 ha during this period, were delineated based on this approach.

#### Field measurement of postfire stand density and aboveground NPP

To determine the vegetation index that serves as the best proxy for recovery, correlations were computed between field measurements and remotely-sensed vegetation indices. Postfire tree recruitment and aboveground net primary productivity (ANPP) at 83 plots were collected in August 2011 from an 8700 ha fire scar burned in June 2000. These plots were stratified by fire severity classes (e.g., low, medium, and high) and aspect (e.g., south-slope, north-slope, and flat). At each plot, a 50 m × 50 m area was selected that was relatively homogenous in vegetation recovery, terrain and burn severity. Three 5 m × 5 m subplots were randomly selected within this area. Species, basal diameter, and height for all tree recruits were recorded within each subplot. The seedling density within the plot was calculated as the average of three subplots. ANPP was calculated using allometric models developed for young saplings of each species^19^,^20^.

### Variable calculation

#### Vegetation indices

Wildfires cause a drastic reduction in the reflectance of visible-to-NIR wavelengths (~0.4 – ~0.9 μm) due to the loss of photosynthetic vegetation, and an accompanied increase in the reflectance of SWIR wavelengths (~1.5 – ~2.3 μm) due to increased char/ash^43^,^46^. Postfire vegetation recovery has the opposite effect on spectral reflectance. To capture the postfire vegetation regrowth processes, four vegetation indices were selected: the Normalized Difference Vegetation Index (NDVI), the Normalized Difference Water Index (NDWI), Tasseled Cap Wetness (TCW), and Tasseled Cap Angle (TCA).

NDVI is a widely used index to quantify the amount of photosynthetically active vegetation or greenness, green vegetation fraction, leaf area index, and net primary production on the ground. As such, the index is also widely used as a proxy for postfire recovery in various ecosystems^25^,^47^. The index is calculated:


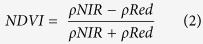


where *ρRed* is the reflectance in the red wavelength, Landsat TM/ETM+ band 3 (0.63–0.69 μm).

NDWI has been shown to be less sensitive to atmospheric scattering and more sensitive to water absorption and canopy structure^26^,^48^, making it potentially suitable to detect structural changes associated with vegetation recovery. NDWI is calculated


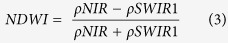


where *ρSWIR*1 is the reflectance in the SWIR wavelength, Landsat TM/ETM+ band 5 (1.55–1.75 μm).

Tasseled Cap (TC) indices are well-known linear transformations of band reflectance and have been widely used for forest mapping and change detection. TC Wetness (TCW) is usually interpreted in vegetated areas as an index of canopy structure, soil or surface moisture, or possibly an estimate of the amount of dead or dried vegetation^49^. Recent analysis has found that TCW is positively associated with woody vegetation cover in the Amazon^50^. Standard TCW transformation coefficients for Landsat bands were derived from the literature^51^.

TCA is defined as the angle formed by TC greenness (TCG) and TC brightness (TCB) in the vegetation plane (Equation 4), representing the proportion of vegetated area to non-vegetated area. TCA has been used to characterize vegetation density and succession in coniferous forests^27^,^52^, and has shown good correlation with LiDAR-derived canopy structure (i.e., canopy cover and height) in a temperate forest in British Columbia, Canada^53^. Denser forest stands show higher TCA values.


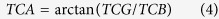


#### Fire severity

Relative dNBR (RdNBR) images for each fire patch were calculated and used as a proxy for fire severity^43^,^44^. RdNBR is calculated by dividing dNBR by the square-root of the prefire NBR. Positive RdNBR values represent a decrease in vegetation cover, just like dNBR, while negative values represent an increase in vegetation cover. RdNBR removes the biasing effect of the prefire conditions on NBR, and makes fire severity comparable among different fires and vegetation types^44^.

Spatial variability of fire severity (RdNBR_Var) and Shape index (SHP_IDX) influence seed availability and delivery because they are representative of residual surviving trees and adjacent unburned forest^11^. The RdNBR_Var was calculated as the standard deviation of RdNBR within a 120 m circle based on the seed dispersal ability of Larch^54^. The SHP_IDX measures the shape complexity and becomes larger as patch shape becomes more irregular. Shape index was calculated as patch perimeter divided by the minimum perimeter possible for a maximally compact patch of the corresponding patch area^55^. Distance to nearest unburned forest (D2B) was included to approximate the effect of fire on seed delivery. Fire year (Year + 0) NDVI was incorporated into the model and used as a proxy for postfire vegetation condition (i.e., remaining vegetation and availability of open space for regeneration).

#### Climate variability

Monthly temperature and precipitation at 0.5 degree resolution from 1986 to 2015 was downloaded from the Climate Research Unit at the University of East Anglia (https://crudata.uea.ac.uk/cru/data/hrg/)^56^. Climate anomalies were used based on the rationale that climate anomalies remove the underlying spatial variability in long-term climate data and emphasize temporal deviations and are therefore effective indicators of regional droughts and other meteorological fluctuations. Monthly climate anomalies were calculated as the difference between monthly climate measurements and long-term (1986–2015) monthly climate measurement averages. The immediate and long-term climate anomalies were summarized to account for the potential effect of climate variability on vegetation recovery at different temporal scales.

Immediate postfire climate conditions influence plant regeneration and establishment via soil moisture conditions and site quality. Immediate postfire climate anomalies were summarized for summer (June, July, August) and winter (December, January, February) at fire years (Year + 0) and one year after fire (Year + 1). Long-term climate conditions influence plant survival and growth rates. Long-term postfire climate anomalies were summarized as the average summer climate anomaly from Year + 0 to Year + 4 when explaining the variation in Year + 5 vegetation recovery. Note that one climate pixel encompassed many 30-meter Landsat pixels, providing a single climate variable value across those 30-meter pixels.

#### Topography

A 30-meter resolution Digital Elevation Model (DEM) for the study area was acquired from the SRTM (Shuttle Radar Topography Mission) website (http://www2.jpl.nasa.gov/srtm/). In this analysis, elevation (m), slope (degree), and potential solar radiation (*Poten_rad*) were used. The *Poten_rad* was used to estimate the potential influence of solar radiation on soil moisture^57^, and is calculated as follows:


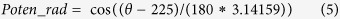


where θ is the aspect derived from the Arc/Info “aspect” function. *Poten_rad* ranged from −1 to 1, with higher values indicating higher potential solar radiation.

### Analysis

#### Exploring vegetation indices as proxies for recovery

To understand which vegetation indices were better indicators of postfire forest recovery, field measurements of seedling density and ANPP were compared with NDVI, NDWI, TCW, and TCA. To minimize the potential spatial mismatch of footprints of field observations and Landsat pixels, values of vegetation indices were averaged within 3 by 3 pixel windows covering the field measurements. Pearson correlations among log-transformed seedling densities, ANPP, and vegetation indices from ETM+ image acquired on August 28, 2011 were calculated. The correlations were done using the ‘pairs.panels’ function in the ‘psych’ package in R^58^.

#### Postfire vegetation recovery trajectory

To explore postfire spectral trajectories, time series of vegetation indices were sampled before and after fires. According to the results of a global Moran’s *I* test on NDWI, the minimum distance between spatial samples required to remove spatial autocorrelation should be at least 1000 meters. The values of vegetation indices at each sampling point were averaged within a 3 by 3 pixel window to remove the potential geolocation bias among images. To focus exclusively on forested cells, only cells with tree cover greater than 30% in 2000 were sampled based on the Landsat-derived Global Forest Change dataset^59^. Then, the mean values of vegetation indices were plotted against time since fire to show the postfire spectral trajectory.

#### Influence of climate variability, fire, and topography on postfire recovery

The spatial locations used to sample time series vegetation indices were also used to sample the values of climate variability, fire characteristics, and topography (predictors). Boosted regression trees (BRT) were used to model vegetation recovery as a function of predictors (Table 1). BRT models are a stochastic extension of classification and regression trees which are “boosted” with additional trees that best reduce the loss function at each step^60^. The BRT method combines the advantages of regression trees, which relate a response to their predictors by recursive binary splits, and boosting algorithms, which combine many simple models to give improved predictive performance. The relative importance and partial dependence of each predictor is calculated in the BRT, along with the interactions between the predictors. The BRT analysis was run in R using the ‘dismo’ package^61^.

The BRT ensemble process is controlled by learning rate (*lr*, the rate at which the model is built), tree complexity (*tc*, the level of variable interactions), and the number of trees (*nt*) to balance model fit and predictive performance. A bag fraction was also used to split the data into the proportion of the data set used to train versus cross-validate the models. In this analysis, predictive deviance, representing the goodness of fit between predicted and observed values, was used to select the best combination of parameters (*lr, tc* and *nt*) using a 10-fold cross validation^61^.

Predictors that had a relative importance greater than 5% were reported, along with their marginal effects on postfire recovery. The BRT model determines the variable importance based on the idea that the relevance of each predictor in a classification tree can be measured as the number of times it is selected for splitting the tree. The relative importance of each predictor was evaluated as the average number of times that the predictor was used in splitting the boosted regression trees, weighted by the squared improvement to the model, averaged over all trees^61^. Partial dependence functions estimate the impact of a given predictor on the dependent variable by statistically controlling for the average effect of all other predictors. The two-way interactive effect of variables was quantified as the variance caused by the two predictors while controlling for the average effect of all other predictors. The joint partial dependence plots were used to provide a clear and easily interpretable depiction of complex interactions^61^.

## Results

### Correlations among field measurements of postfire stand density, ANPP, and vegetation indices

**Figure 4:**
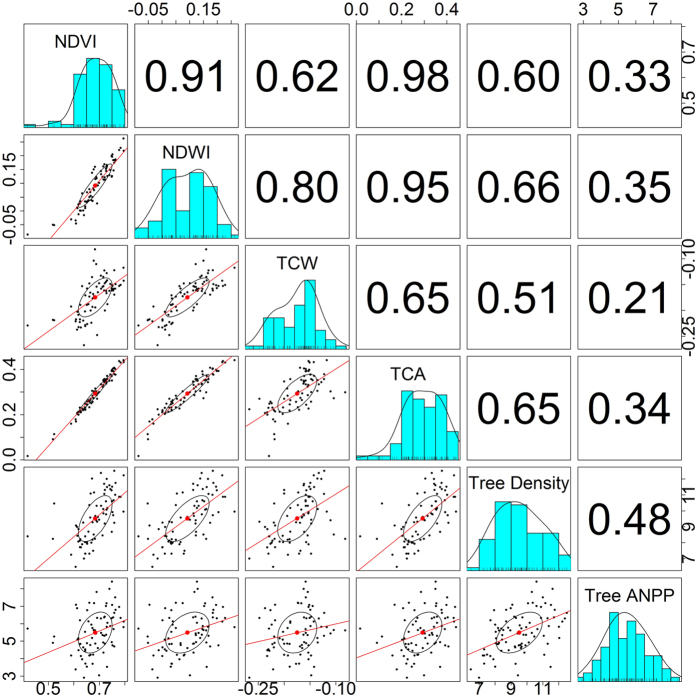
Correlation among field measurement of stand density and ANPP, and different vegetation indices. Circle represents correlation ellipses among variables. NDVI: Normalized Difference Vegetation Index, NDWI: Normalized difference water index, TCW: Tasseled Cap wetness, TCA: TC Angle.

As expected, vegetation indices were highly correlated with each other, but the strength of the correlation decreased as the vegetation indices used different proportions of each wavelength (Fig. 4). All vegetation indices had a stronger relationship with tree density than with ANPP, suggesting a decoupling between ecosystem structure and function, and remotely sensed vegetation indices. The NDWI had the strongest relationship (*R*^2^ = 0.66) with tree density, while the TCW had the weakest relationship (*R*^*2*^ = 0.51). The widely-used NDVI ranked third in terms of correlation with tree density (*R*^2^ = 0.60). Density plot indicated that these field measurements are representative of fire severity and postfire vegetation indices across the study area, despite they are mainly from high elevations (Supplementary Fig. S1). As such, NDWI is the most robust proxy for postfire vegetation recovery in boreal Larch forests of Northeastern China.

### Postfire vegetation recovery trajectory

**Figure 5:**
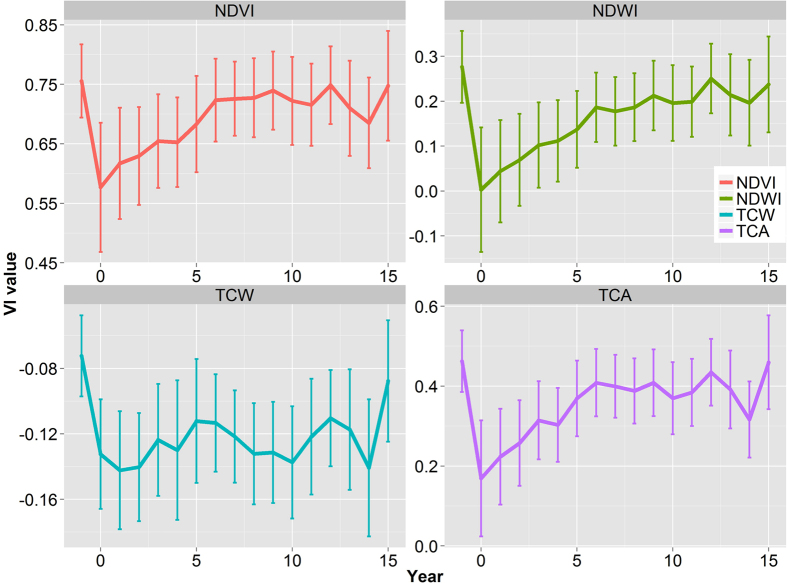
Postfire trajectories of vegetation indices in the study area. Error bar stands for plus-or-minus standard deviation. NDVI: Normalized Difference Vegetation Index, NDWI: Normalized difference water index, TCW: Tasseled Cap wetness, TCA: TC Angle.

After controlling for spatial autocorrelation and excluding non-forest pixels, a total of 13,112 pixels were sampled to examine postfire vegetation recovery trajectories. The four selected vegetation indices dropped dramatically after fire disturbance, and such drops were larger for NDWI and TCA (Fig. 5). All vegetation indices, except for TCW, increased monotonically until 5–6 years after fires, but become more variable thereafter. TCW did not show a clear trend after fires. Five years after fire, the NDVI, NDWI, and TCW were not statistically significantly different from pre-fire levels. Such a rate of spectral recovery is comparable to that from *Larix sibirica* in northern Mogolia and mixed-conifer forests of the Sierra Nevada, California, USA^47^,^62^, but much faster than that from Larch forests in Central Siberia, Russia^25^. As a result, vegetation indices 5 years following fire (Year + 5 VI) were chosen as a proxy for short-term vegetation recovery.

### Influences of climate, fire, and topography on postfire vegetation recovery

**Figure 6:**
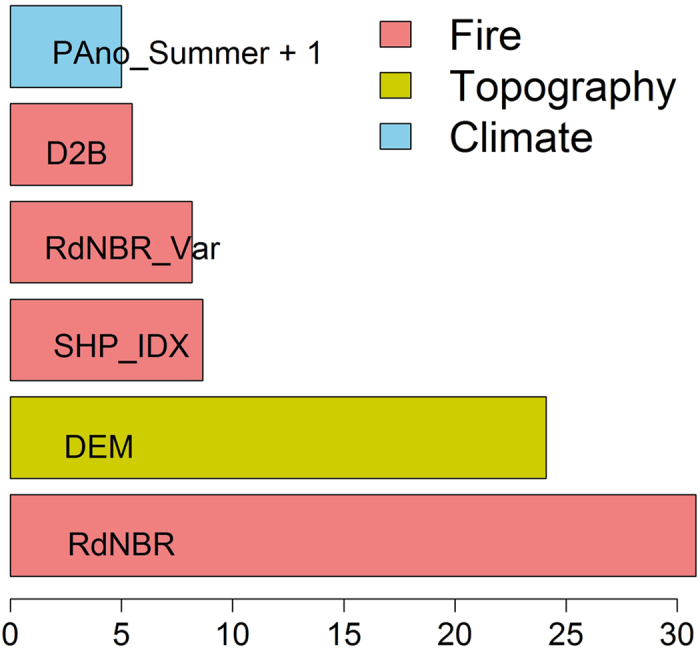
Relative influences of variables that explained greater than 5% of the variation from boosted regression tree models of vegetation recovery. For explanation of variables and their units see Table 1.

**Figure 7:**
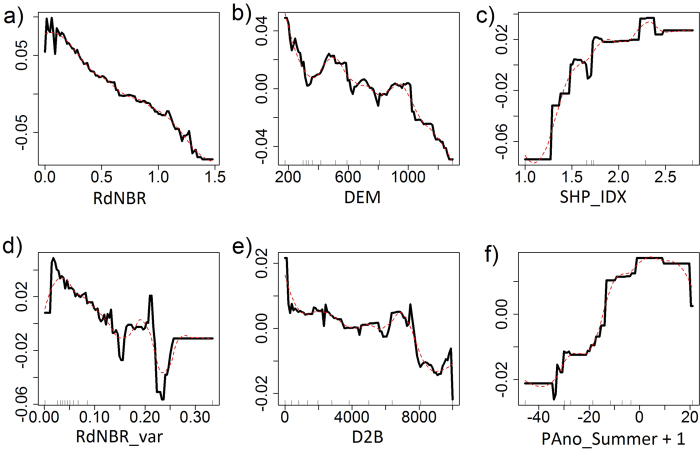
Partial dependency plots for variables in a boosted regression tree predicting postfire vegetation recovery. Partial dependency plots represent the estimated marginal effect of a variable on postfire recovery when all other variables are held at their average. The y-axis indicates the relative effects of a variable (x-axis) on vegetation recovery. Red lines indicate the marginal effects constrained in the model to be monotonic. Tick marks at the x-axis indicate the deciles (10% quantiles) of the observed distribution of continuous predictor variables. For explanation of variables and their units see Table 1.

**Figure 8:**
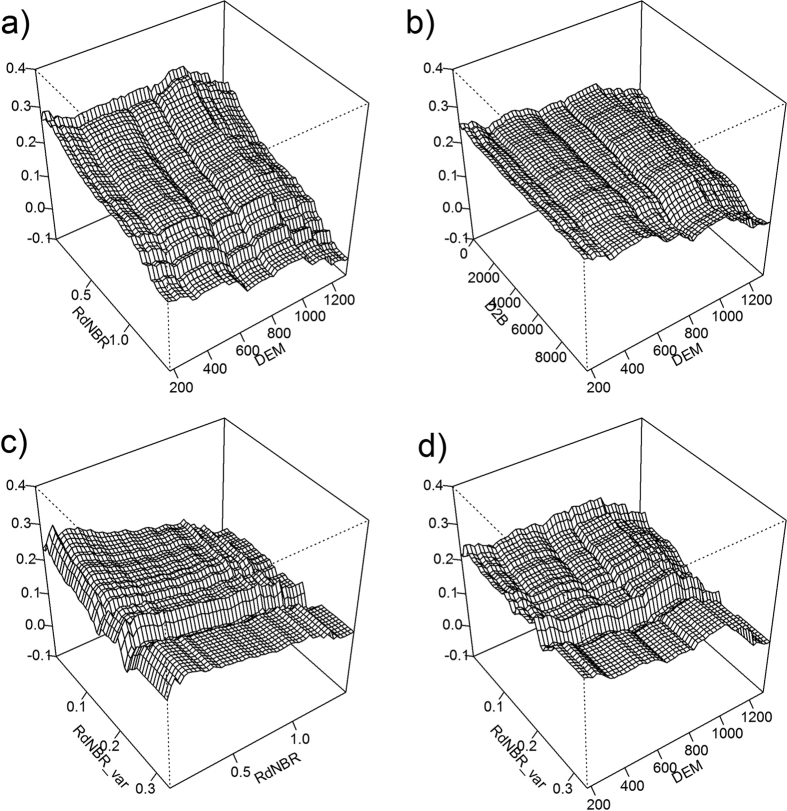
Three-dimensional partial dependence plots for the top four strongest interactions in the model for postfire vegetation recovery. All variables except those graphed are held at their means. The z-axis indicates the relative effects of interactions between variables (x- and y-axis) on vegetation recovery. For explanation of variables and their units see Table 1.

Based on the previous analysis, NDWI at 5 years after fire represents a robust proxy for short-term vegetation recovery due to its strong correlation with field measurements of stand density (Figs 4 and 5). The best BRT model, with a sample size of 13,112, explained 64.8% of variance with *tc* = 3, *nt* = 900, *lr* = 0.1, and a bag fraction of 0.5. There was no strong multi-collinearity among predictors. The BRT analysis showed that fire characteristics, climate variability, and environmental filters all had significant influences on short-term vegetation recovery (Fig. 6). Fire severity had the strongest influence on postfire recovery, followed by elevation, shape index, spatial variability of fire severity, distance to unburned forest, and summer precipitation one year after fire. Partial dependencies from the fitted model indicate that, when other variables were held constant, vegetation recovery was negatively correlated with fire severity, spatial variability of fire severity, elevation, and distance to unburned forest, and positively correlated with shape complexity and summer precipitation one year after fire (Fig. 7). Forcing the marginal effects in the model to be monotonic had little effect on the overall shape of the estimated effects of predictors on vegetation recovery. The strongest interaction was found among fire severity, spatial variability of fire severity, elevation, and distance to unburned patches. Vegetation recovery was faster in sites where fire severity and its spatial variability was lower, closer to unburned patches, and at lower elevations (Fig. 8).

## Discussion

### Remote sensing of postfire vegetation recovery

The results show that ecosystem structure is more strongly correlated with SWIR-based vegetation indices (e.g., NDWI) than with Red/NIR-based vegetation indices (NDVI), suggesting that SWIR-based vegetation indices are better indicators for postfire vegetation recovery in boreal Larch forests. Previous analyses also showed that SWIR-based vegetation indices are sensitive to the interaction between water content and canopy structure in boreal^27^, temperate^48^, and tropical forests^26^,^63^. This is because the spectral reflectances at SWIR wavelengths are sensitive to canopy water content and vegetation structure, and also have large dynamic changes^64^. The Red/NIR-based vegetation indices are mainly related to “greenness”, which reflects the horizontal surface area of photosynthesizing vegetation. Therefore, SWIR-based vegetation indices are sensitive to vegetation structure and dynamics^25^,^65^, and provide good indicator of forest recovery.

### Spatial controls of postfire vegetation recovery

Fire severity and its spatial variability played a dominant role in determining potential vegetation recovery following fire in this Siberian boreal larch forest. Environmental filters appear to be a weaker force, but interacted strongly with fire severity to influence postfire recovery. Immediate postfire climate conditions play a minor, but significant role in vegetation recovery. This analysis thus revealed that postfire recovery in boreal Larch forests of Northeastern China was collectively controlled by processes that controlled seed availability, site condition, and climate variability after fire. Anticipated increases in fire severity and moisture stress^66^, interacting with gradients of environmental conditions, will have significant influences on vegetation structure and function, and forest feedbacks to climate^67^,^68^.

Previous research has demonstrated that fire determines vegetation recovery through its influence on seed availability and site conditions, and species’ life-history traits. For example, in fire “embracer”-dominated forests, consumption of organic soil layers favors deciduous hardwood regeneration over coniferous regeneration and thus alters successional trajectories after fire^9^. In fire “avoider”-dominated forests, deciduous hardwoods are generally less dispersal-limited (due to their relatively long dispersal distances) than evergreen conifers (shorter dispersal distances). Thus, the landscape-scale balance between deciduous hardwoods and evergreen conifers in terms of seed limitation is the primary mechanism for successional trajectory changes after fire^13^. In a ‘resister’-dominated (e.g., larch) forests, postfire recovery is generally not considered seed-limited because it frequently survives fire due to its species’ thick bark, which has a low thermal diffusivity. Contrary to the expectation, this analysis suggested that seed availability is likely the primary mechanism to limit postfire forest recovery in the larch forests of Northeastern China, because the thin organic soil layer (<10 cm) is usually completely consumed by fires^19^,^20^.

The seed limitation on postfire recovery was reaffirmed by the fact that the spatial variability of fire severity also had significant negative effects on vegetation recovery, potentially due to its effect on vegetation legacies and seed availability. Indeed, vegetation legacies after fire affect seed availability and play a pivotal role in shaping patterns of community assembly after fire in these low-diversity boreal forests^12^,^13^. As Larch forests are becoming increasingly seed-limited in response to intensifying fire regimes, lack of seed may potentially change forests to alternative vegetation-disturbance feedbacks under a future warmer and drier climate (e.g., shrubland or grassland with frequent, high-severity, fires), consistent with conclusions from a field-based study^20^.

Climate conditions in the first postfire growing season play an important role for vegetation recovery, consistent with a recent analysis in the Sierra Nevada^47^. Specifically, higher growing season precipitation one year after fire has a positive influence on postfire recovery, while temperature and longer-term climate conditions have no significant effects. This suggests that recovery of plant demography (i.e., colonization and establishment) is strongly controlled by moisture availability. The effect of moisture availability on initial postfire colonization and establishment is more critical than longer-term forest growth on vegetation recovery. Similar results have been found in many other ecosystems^17^,^47^,^69^. As the frequency and severity of drought is expected to increase under a warmer and drier climate, elevated chances of postfire water stress may have a significant influence on ecosystem composition and function^20^. Recent climate warming has resulted in a rather uniform trend toward higher plant productivity and forest cover gain in the North, and a trend toward decreased productivity and forest cover loss in southern Siberian boreal forests^66^, similar to observations from Canada’s boreal forests^70^.

Understanding the interactive effects between fast-changing fire regimes and slow-changing climate on ecosystem change in Siberian larch forests is critical for anticipating its potential role in Earth system. Unlike the typical boreal fire regime in North America, the fire regime in Siberian Larch forests is characterized by early season (March–May), low-intensity surface fires, mixed with infrequent stand-replacing fires under extreme climate conditions^5^,^71^,^72^,^73^. If an intensified fire regime unfolded as expected under warmer and drier climate conditions, accelerated changes in species composition or even biome boundaries may potentially hasten transitions to ‘no-analogue communities’ in the region^74^. If large-scale changes in biotic communities occur after disturbance, there will also be significant consequences for many ecosystem processes, such as carbon cycling and thawing of the permafrost^66^. Therefore, the potential interactions among climate, fire, and ecosystem change in Siberian Larch forests warrant further research.

### Uncertainties

The climate data used had a much coarser resolution compared to topography and fire data. This may have resulted in a decreased explanation of spatial variation and an underestimation of the influence of climate on vegetation recovery. To assess such an effect, a BRT analysis was conducted at the fire patch level (n = 84), with *tc* = 3, *nt* = 240, *lr* = 0.01, and bag fraction = 0.75. This model explained 84.8% of the variation in vegetation recovery, and confirmed that fire severity had the strongest influence on the variable, followed by elevation, and both had negative correlations with vegetation recovery (Supplementary Fig. S2). Postfire climate variability explained less than 5% of the variation. Because topography and associated soil moisture may influence vegetation recovery, three additional BRT models were fit, stratified by potential solar radiation. All three models suggested that the major conclusions of the original analysis were robust across different topographic positions and different scales (Supplementary Fig. S3). This showed that the scale mismatch between climate and environmental variables did not change the main findings of this analysis, possibly due to the following two reasons. First, Moran’s *I* was used to exclude the spatial structures of variables. Second, climatic anomalies were used to remove the underlying spatial variability in long-term climate and emphasize temporal fluctuation in climatic condition at local scales.

Although time series analyses of remotely sensed data provides a method to monitor forest recovery and vegetation reestablishment following disturbances^75^,^76^,^77^, the linkage between spectral recovery and ecological or silvicultural understanding of forest recovery has rarely been made^78^. From an ecological perspective, forest recovery is typically considered as the reestablishment of forest biomass or canopy structure following disturbance and may be indicated by vegetation structural properties, photosynthetic capacity, height, biomass, and heterogeneity^79^. Therefore, the vegetation recovery in this analysis should be interpreted as spectral recovery, rather than structural or functional recovery from an ecological perspective. In the future, multi-sensor approaches combining airborne LIDAR with satellite data may link sensor data to actual forest structure and biomass data at fine resolution over large spatial scales.

Vegetation recovery is a multi-faceted process. Nevertheless, most studies have primarily focused on investigating recovery of photosynthetically active vegetation or “greenness” by remote sensing approaches. However, spectral recovery does not necessarily reflect recovery in other ecosystem variables such as biogeochemistry, radiation budgets, or water balance. Multiple recovery indicators should be used as a way to provide complementary measurements of vegetation dynamics, and help trend interpretation and attribution of proximate causes.

## Conclusions

Two key mechanisms governing postfire recovery in boreal forests are seed availability related to fire characteristics and seedling establishment/survival related to site and postfire climatic conditions^16^. Testing the interactive effects of climatic conditions and fire characteristics in controlling postfire recovery across multiple burned patches provides an indicator of forest resilience to future climate and disturbance in boreal forests. This analysis revealed that postfire recovery in boreal Larch forests of Northeastern China was primarily controlled by seed availability in relation to fire severity and its spatial variability. Once seeds arrive, successful germination and seedling establishment are governed by moisture conditions and local site factors. This study suggested that forest resilience in the Siberian Larch region was collectively controlled by processes that controlled seed availability, as well as by site conditions and climate variability. If future drought and fire regimes manifest as expected in this region, the seed limitation and climate-induced regeneration failure will become more prevalent and severe, which may cause widespread forest dieback and compositional change. More research is needed to address the degree to which climate change-induced intensification of fire regimes will cause forests to shift to alternative stable states, as well as how changes in fire regimes may impact regional biogeochemistry, energy, and water cycles.

## Additional Information

**How to cite this article**: Liu, Z. Effects of climate and fire on short-term vegetation recovery in the boreal larch forests of Northeastern China. *Sci. Rep*. **6**, 37572; doi: 10.1038/srep37572 (2016).

**Publisher’s note:** Springer Nature remains neutral with regard to jurisdictional claims in published maps and institutional affiliations.

## Supplementary Material

Supplementary Information

## Figures and Tables

**Table 1:** predictors used to quantify the influence of climate, fire, and topography on postfire vegetation recovery in the boosted regression tree model.

Variables	Descriptions and related mechanisms in Fig. 2.	Hypothetical relationship with vegetation recovery	Range (median)
Year + 5 NDWI (unitless)	A proxy for postfire vegetation recovery status, 5-year postfire		−0.43–0.53 (0.20)
RdNBR (unitless)	A proxy for fire severity, Relative dNBR (M1 & M3).	Negatively, higher fire severity consumes more seed stored in soil and canopy and reduces seed availability^19^,^20^	0–1.48 (0.36)
RdNBR_var (unitless)	A proxy for spatial variability of fire severity, standard deviation of RdNBR within a 120 m radius (M1)	Positively, higher spatial variability of fire severity increases the chance of tree survival and provides more seed within burned patches^11^	0.00117–0.334 (0.0455)
D2B (m)	A proxy for seed dispersal distance, distance to nearest unburned forest (M2)	Negatively, the probability of seed reach to a site decreases exponentially with distance to seed sources	0–9999 (2790)
SHP_IDX (unitless)	A proxy for fire patch characteristics, shape index for each fire (M2).	Positively, higher patch complexity increase the probability of seed reach to a site from the seed source	1–2.79 (1.71)
TAno_Summer + 0 (degree)	Summer temperature anomaly for the fire year (M4).	Unimodal, plants regenerate and grow better at the intermediate level of temperature^17^	−0.88–1.48 (−0.83)
TAno_Winter + 0 (degree)	Winter temperature anomaly for the fire year (M4).	The same as TAno_Summer + 0	−4.41–2.88 (0.39)
TAno_Summer + 1 (degree)	Summer temperature anomaly one year after fire (M4).	The same as TAno_Summer + 0	−0.30–0.99 (0.30)
TAno_Summer + 1–5 (degree)	Average summer temperature anomaly from one to five years after fire (M5).	The same as TAno_Summer + 0	−0.039–0.59 (−0.0089)
PAno_Summer + 0 (mm)	Summer precipitation anomaly for the fire year (M4).	Positive, plants regenerate and grow better at higher moisture availability^17^	−45.41–58.95 (36.97)
PAno_Winter + 0 (mm)	Winter precipitation anomaly for the fire year (M4).	The same as PAno_Summer + 0	−1.94–4.43 (0.55)
PAno_Summer + 1 (mm)	Summer precipitation anomaly one year after fire (M4).	The same as PAno_Summer + 0	−45.41–21.08 (−11.54)
PAno_Summer + 1–5 (mm)	Average Summer precipitation anomaly from one to five years after fire (M5)	The same as PAno_Summer + 0	−21.63–9.83 (−7.16)
DEM (m)	Elevation	Negative, higher elevation is often associated with higher fire severity and lower soil moisture, and decreases forest recovery^19^,^20^	177–1297 (416)
Slope (degree)	Slope	Negative, steeper slope has thinner soil layer and lower soil moisture, and decreases forest recovery^19^,^20^	0.000–17.3 (3.168)
Potential radiation (unitless)	A proxy for solar radiation and soil moisture	Negative, higher solar radiation decrease soil moisture, and decreases forest recovery	−1–1 (−0.099)
