# Supplementary material for: Effects of climate and fire on short-term vegetation recovery in the boreal larch forests of Northeastern China
Source: Sci Rep. 2016 Nov 18;6:37572. doi: 10.1038/srep37572 (PMC5114605; doi:10.1038/srep37572)
Supplement: Supplementary Information [file srep37572-s1.pdf]

Supplementary Information

Effects of climate and fire on short-term vegetation recovery in the boreal Larch forests of Northeastern China

Zhihua Liu

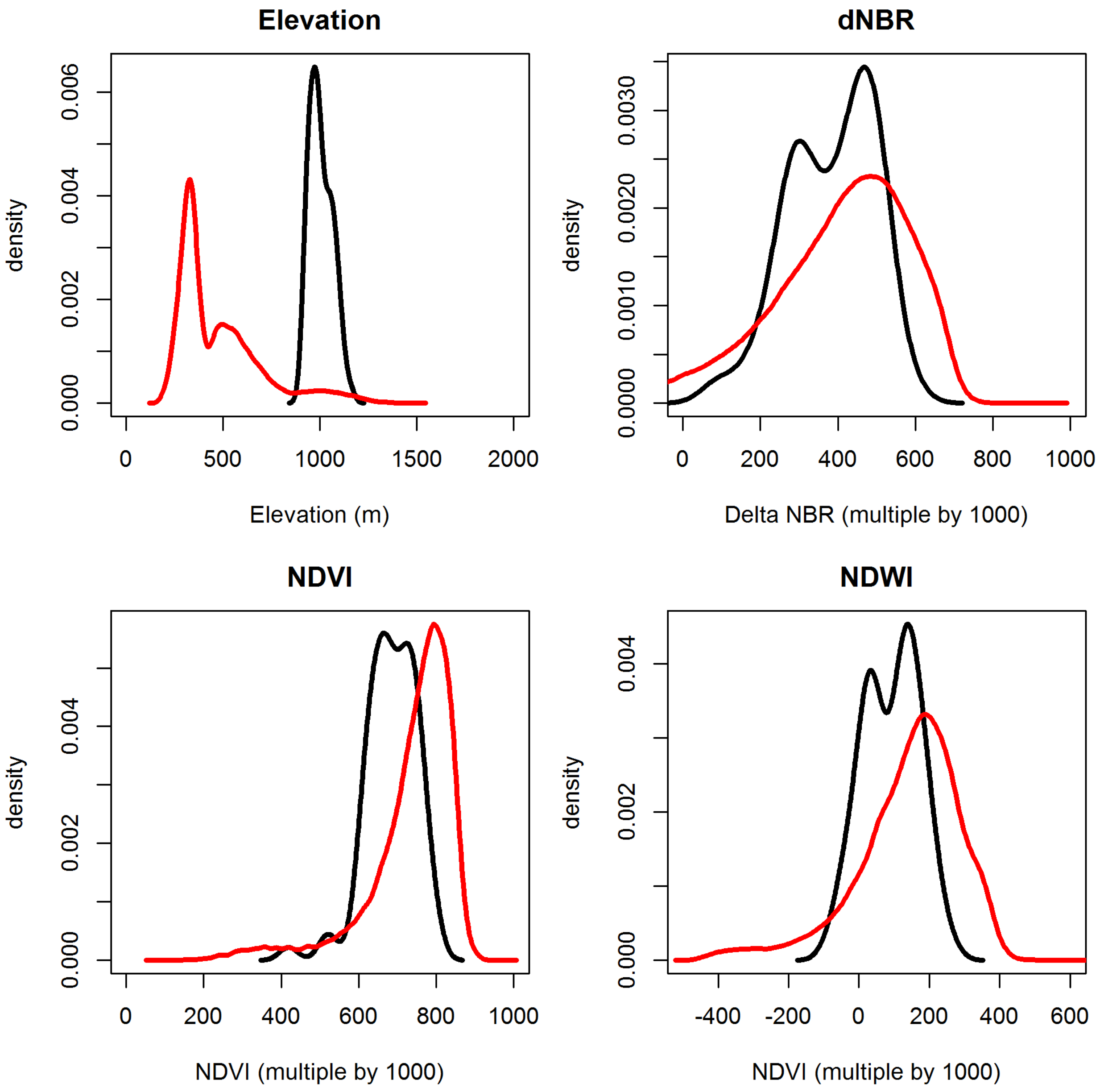

Fig. S1. Density distribution of (a) elevation, (b) dNBR, (c) NDVI, and (d) NDWI between filed sampling site (n = 83, black line) and all the fires (red line). See main text for abbreviations.

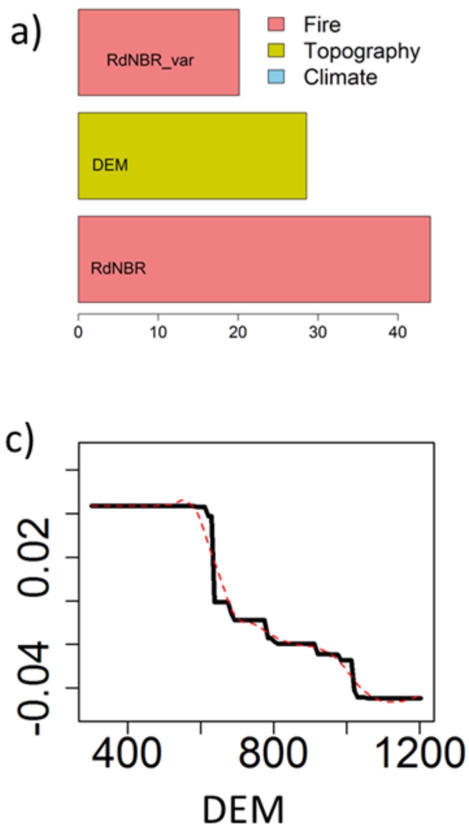

Fig. S2. Relative influence (a) and partial dependency plots (b-d) for top three variables (> 5% of relative influence) in a boosted regression tree (BRT) model predicting postfire vegetation recovery at the fire patch level (n = 84). This BRT model (*tc* = 3, *nt* = 240, *lr* = 0.01, and bag fraction = 0.75) explained 84.8% of the variation. See main text for abbreviations.

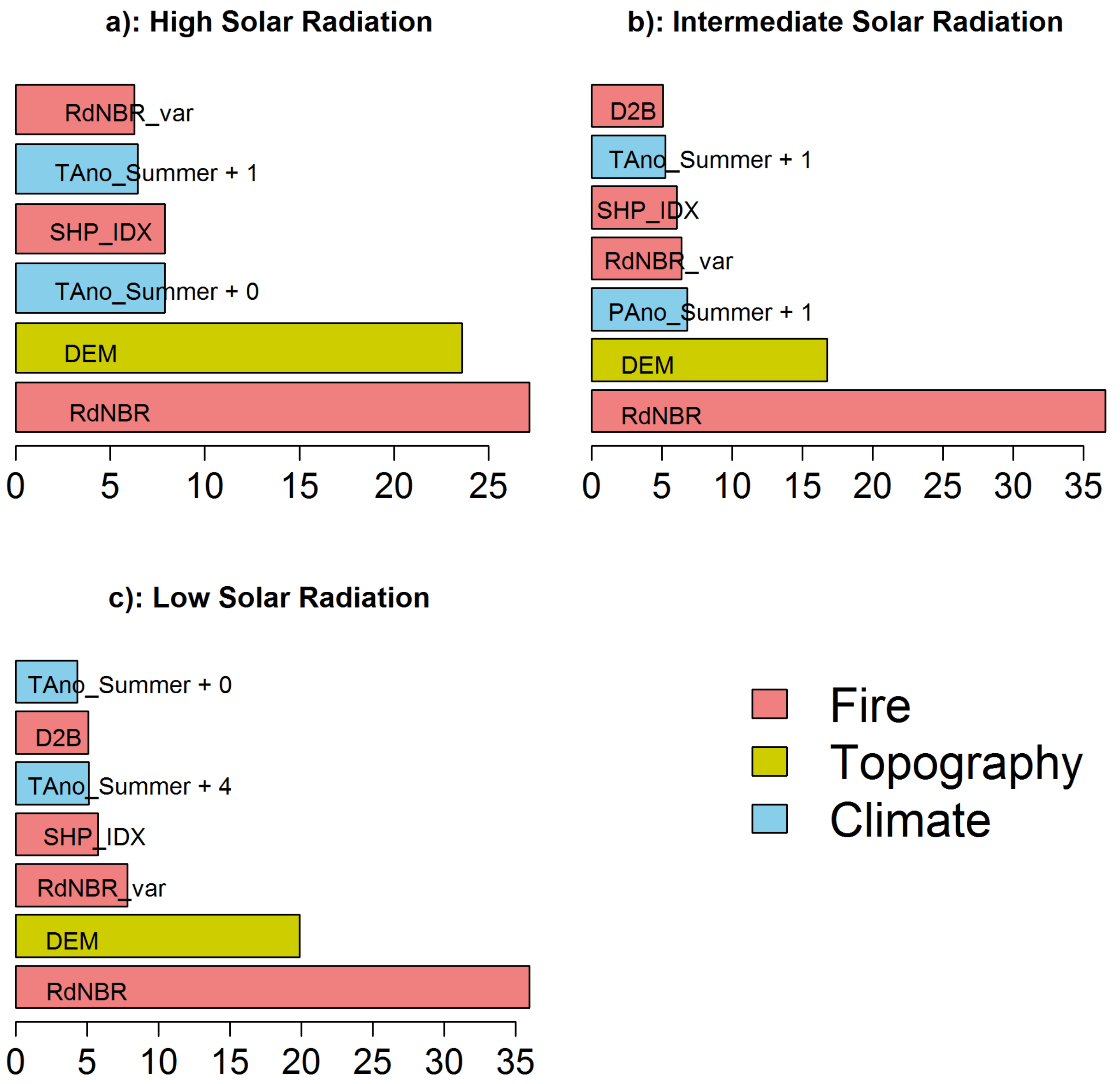

Fig. S3. Relative influence of variable (> 5% of relative influence) on postfire vegetation recovery predicted by boosted regression tree (BRT) models, stratified by potential solar radiation. The high solar radiation (> 0.3) BRT model (*tc* = 3, *nt* = 920, *lr* = 0.1, and bag fraction = 0.75) explained 72.2% of the variation. The intermediate solar radiation (-0.3 - 0.3) BRT model (*tc* = 3, *nt* = 440, *lr* = 0.1, and bag fraction = 0.75) explained 71.8% of the variation. The low solar radiation (< -0.3) BRT model (*tc* = 3, *nt* = 820, *lr* = 0.1, and bag fraction = 0.75) explained 68.9% of the variation. See main text for abbreviations.
